# Evaluation of natural porcine circovirus type 2 (PCV2) subclinical infection and seroconversion dynamics in piglets vaccinated at different ages

**DOI:** 10.1186/s13567-016-0405-2

**Published:** 2016-12-03

**Authors:** Salvador Oliver-Ferrando, Joaquim Segalés, Sergio López-Soria, Antonio Callén, Olivier Merdy, François Joisel, Marina Sibila

**Affiliations:** 1 IRTA, Centre de Recerca en Sanitat Animal (CReSA, IRTA-UAB), Campus de la Universitat Autònoma de Barcelona, 08193 Bellaterra, Spain; 2 UAB, Centre de Recerca en Sanitat Animal (CReSA, IRTA-UAB), Campus de la Universitat Autònoma de Barcelona, 08193 Bellaterra, Spain; 3 Departament de Sanitat i Anatomia Animals, Facultat de Veterinària, UAB, 08193 Bellaterra, Spain; 4 Merial Laboratorios, 08014 Barcelona, Spain; 5 Merial S.A.S., 69007 Lyon, France

## Abstract

This study aimed to determine the porcine circovirus type 2 (PCV2) serological and virological dynamics in piglets vaccinated at different ages in a PCV2 subclinical infection (PCV2-SI) scenario. Six hundred and forty-four 2 week-old healthy piglets were selected and distributed into four treatment groups: vaccination at 3, 6 or 10 weeks of age (3W-VAC, 6W-VAC and 10W-VAC groups, respectively) and unvaccinated pigs (NON-VAC group). Blood (*n* = 112 pigs) and oral fluid (OF) (*n* = 40 pens) samples were taken throughout the study to assess PCV2 load, humoral immunity and viral genotyping. Percentage of PCV2-DNA positive sera mainly raised by 10 weeks of age, being maximum at 14 weeks of age, and then started to decrease at 18 and 25 weeks of age. Specifically, PCV2 vaccination at 3 or 6 weeks of age yielded similar results, since they produced an earlier seroconversion and reduced, at different sampling points, the proportion of viremic animals in comparison to the unvaccinated group. In contrast, PCV2 vaccination at 10 weeks of age only achieved such reduction at 25 weeks of age; in this case, vaccination coincided with the increase of the percentage of viremic pigs in the population. Both serological techniques used in sera and OF offered similar results with a high and statistically significant correlation. In contrast, a higher percentage of PCV2 DNA positivity was detected in OF in comparison with sera. In conclusion, under the present study conditions, the optimal time for PCV2 piglet vaccination was at either 3 or 6 weeks of age.

## Introduction

Porcine circovirus type 2 (PCV2) is one of the most prevalent viruses that cause great economic losses to the worldwide pig industry [1]. This virus has an ubiquitous nature in the pig population and is the causative agent of a number of clinical and subclinical conditions named Porcine circovirus diseases (PCVDs) [2].

Efficacy of PCV2 commercial vaccines has been widely demonstrated under field conditions. Major effects have been seen on the reduction of the impact of PCV2 systemic disease (PCV2-SD), improvement of production parameters, decrease of co-infections, and reduction of PCV2 viremia and shedding [3]. Indeed, the wide and constant use of such vaccines in PCV2-SD scenarios has led, in most farms, to a PCV2 subclinical infection (PCV2-SI) scenario [4]. Moreover, most of the pigs from PCV2-SD affected farms but not displaying this condition also suffer from a PCV2-SI. Furthermore, vaccination against PCV2 has been shown to be economically worthy even in PCV2-SI scenarios [5–8].

The most common age of piglet vaccination against PCV2 is at 3–4 weeks of age (around weaning). However, when a sow and piglet vaccination strategy is planned, a delayed piglet vaccination should be considered in order to achieve higher vaccine efficacy [9]. Under no vaccinated sow scenario, little information is available whether the 3–4 week vaccination-age offers the best profit. Although PCV2 vaccines are routinely used in most of the worldwide porcine production systems, peer-reviewed studies comparing the efficacy of PCV2 vaccination at different ages are scarce in experimental [10, 11] and, particularly, under field conditions [12].

Serum is the most commonly used sample to assess PCV2 antibody and genome detection [2]. However, blood sampling is an individual and invasive method. Oral fluid (OF) is an economic and easy-to-take sample for detecting antibodies and pathogens in a pig population [13–15]. This fact allows a more frequent herd monitoring and a greater representativeness of the animal group. During last years, PCV2 dynamics after natural [16, 17] or experimental [18] infections has been efficiently monitored by OF samples, using both enzyme-linked immunosorbent assay (ELISA) and polymerase chain reaction (PCR) techniques. However, information regarding PCV2 assessment in terms of viral load and antibody levels in OF after immunization is limited [19].

The objectives of the present study were: (a) to determine the optimal time for PCV2 vaccination, in terms of serological and virological parameters, in pigs vaccinated at 3, 6 or 10 weeks of age in a PCV2-SI scenario under common PCV2 circulation timings, and (b) to expand the knowledge on the use of OF samples to detect PCV2 DNA and antibodies.

## Materials and methods

### Farm selection

The study was conducted in a conventional pig farm, located in Catalonia (Spain). In order to assess PCV2 infection status before the start of the study, a cross-sectional seroprofiling was performed including 10 pigs per batch of 6 age groups (3, 7, 11, 15, 19 and 23 weeks of age). Blood samples were processed by standard PCR [20] and ELISA (Ingezim Circo IgG 11.PCV.K1®, Ingenasa, Madrid, Spain) to detect viral nucleic acid and antibodies (IgGs), respectively. PCV2 genome was detected in 50, 30, 20 and 10% of the sampled pigs at 11, 15, 19 and 23 weeks of age, respectively. All the 3 and 7 weeks tested samples were negative by PCR. Seroconversion was detected from the 11th week of age onwards. Therefore, as no PCVDs clinical signs were evident in the farm, PCV2-SI was confirmed.

This farm was a two-site herd with 800 sows with all-in/all-out management and 4-week batch farrowing system. PCV2 vaccination in sows and piglets had never been applied in the studied herd. Sows were routinely vaccinated against porcine reproductive and respiratory syndrome virus (PRRSV), Aujeszky’s disease virus, Swine influenza virus, porcine parvovirus, *Erysipelothrix rhusiopathiae*, *Escherichia coli* and *Clostridium perfringens.* Piglets were vaccinated against *Mycoplasma hyopneumoniae* 3 days pre-weaning. Weaning was performed at 3 weeks of age and pigs were moved to fattening units at 10 weeks of age. Moreover, no signs of any major pig diseases were present and herd immunity status against PRRSV was determined as “positive-stable” (II-A) according to the previously described classification [21].

### Study design

Six-hundred and forty-four 2 week-old healthy crossbred piglets were selected in one single farrowing batch. These piglets came from 59 PCV2 non-vaccinated sows with low number of weak and cross-fostered piglets in their litters. Piglets were individually identified (ear-tagged), bled and their gender was recorded. Blood samples were tested by ELISA (Ingezim Circo IgG 11.PCV.K1®). Cross-fostered piglets were not included in the trial. At 3 weeks of age, animals were randomly allocated in four treatment groups (Table 1). Groups were randomized according to PCV2 ELISA S/P values, sex and litter. Animals from different treatment groups were housed in different pens (32 pens in nursery and 56 pens in fattening units) following a chessboard pattern. Pigs were vaccinated by intramuscular injection with 0.5 mL (single dose) of a commercial inactivated PCV2 vaccine (CIRCOVAC®, Merial SAS, Lyon, France) at either 3, 6 or 10 weeks of age (3W-VAC, 6W-VAC and 10W-VAC groups, respectively), and another group of pigs was kept unvaccinated (NON-VAC group).

**Table 1:** **Experimental design**

Group	Total number of pigs	Number of bled pigs	Number of pens tested by OF samples		Treatment		
			Nursery unit^a^	Fattening unit^b^	3 weeks of age	6 weeks of age	10 weeks of age
3W-VAC	161	28	6	10	Vaccination^c^	–	–
6W-VAC	161	28	6	10	–	Vaccination^c^	–
10W-VAC	161	28	6	10	–	–	Vaccination^c^
NON-VAC	161	28	6	10	–	–	–

^a^Approx. 23 pigs were allocated in each nursery pen.

^b^Approx. 11 pigs were allocated in each fattening pen.

^c^Animals were vaccinated with a single dose (0.5 mL) of CIRCOVAC® (Merial SAS, Lyon, France).

Among all animals included in the study, 28 animals per group (14 males and 14 females) with a medium antibody titre (ranging from 0.07 to 1.24 ELISA S/P values at 2 weeks of age) and equally distributed in all pens (2 or 4 piglets per pen in nursery and 2 piglets per pen in fattening units) were randomly selected to be bled. From these animals, a blood sample was taken at 6, 10, 14, 18 and 25 weeks of age. Whole blood samples were allowed clotting, and centrifuged at 3200 rpm during 20 min at 4 °C. All sera were aliquoted and stored at −20 °C until testing.

Oral fluid samples were collected from a representative number of pens (24 nursery and 40 fattening pens) located at the entrance, middle and final area of the nursery and fattening units. OF were taken simultaneously to blood collection by suspending a non-treated, 3-strand, 100% cotton rope in each pen for 30 min [22]. Each rope was manually squeezed inside a single-use plastic bag for OF extraction; then, the corner of the bag was cut and the sample was poured into a sterile tube. To avoid cross-contaminations, all materials (bag, globes, tube) were changed or disinfected (scissors) between pens. Once in the laboratory, samples were centrifuged at 1000×*g* during 10 min at 4 °C for clearing the sample [14]; then, the supernatant was aliquoted and frozen at −80 °C until use.

From 2 to 25 weeks of age (at each vaccination or bleeding point), all pigs included in the study were monitored for clinical signs and mortality. Animals with major pathologies (hernia, lameness, injuries, etc.) were excluded from the study. Housing conditions, feeding system, feed characteristics and health management remained consistent along the course of the trial, and were the same among all experimental groups. The present study was approved by the Ethics Committee for Animal Experimentation from the *Universitat Autònoma de Barcelona* and the Animal Experimentation Commission from the local government (*Dpt. de Medi Ambient i Habitatge* from the *Generalitat de Catalunya*; Reference 5796).

### DNA extraction and real-time quantitative PCR in serum and OF samples

DNA was extracted from 200 µL of serum or 300 µL of OF samples, by using the MagMAX™ Pathogen RNA/DNA Kit (Applied Biosystems) following the manufacturer’s instructions. The DNA obtained was suspended in 90 µL of elution solution.

To quantify the PCV2 DNA in serum and OF samples, a real-time qPCR assay (LSI VetMAX™ Porcine Circovirus Type 2 Quantification, Life Technologies) was performed. Each extraction and qPCR plate included negative controls (diethylpyrocarbonate (DEPC)-treated water) and each sample reaction had an internal positive control (IPC) to monitor DNA extraction and amplification procedures. Viral concentrations were expressed as the mean log_10_ PCV2 genome copies/mL. Area under the curve (AUC) of viral load in serum samples from 2 to 25 weeks of age was calculated according to the trapezoidal method as previously described [23].

### Serology

#### Indirect ELISA for detecting anti-PCV2 IgG antibodies in serum samples

All serum samples were tested by the Ingezim Circo IgG 11.PCV.K1® assay. The optical density (OD) was measured at 450 nm by the PowerWave XS reader (BioTek). Mean positive cut-off was established at 0.3 OD (±SD) following the kit’s instructions (positive cut-off = OD of negative control +0.25). ELISA results were expressed as mean S/P ratio (OD of sample/OD of positive control for each ELISA plate).

#### Semi-quantitative ELISA for detecting anti-PCV2 antibodies in OF samples

All OF samples were processed by the SERELISA® PCV2 Ab Mono Blocking kit (Synbiotics, Lyon, France) with some modifications (protocol used at Labocea, Ploufragan—personal communication). The analysis of the samples by this technique led to a semi-quantitative result expressed as 1 (+), 2 (++), 3 (+++) or 4 (++++).

#### Viral neutralization test (VNT)

The ability to neutralize PCV2 was assessed by VNT in 14 randomly selected serum samples per group (half of collected serum samples). This assay was performed as previously described [24], with the following modifications: (1) serum was tested in fourfold dilutions (from 1:4 to 1:4096) using supplemented DMEM (Dulbecco’s Modified Eagle Medium) in 96-well plates (plates were read using a microscope at 10× magnifications), and (2) number of PCV2 infected cells (nuclear and/or cytoplasmic staining) per well in each sample replica was counted. Percentage of virus neutralization (%VN) at each serum dilution was calculated as follows:  % VN = [1 − (mean number of infected cells of the two replicas of each serum dilution/mean number of infected cells in negative control wells)] ×100. Then, VNT50 (i.e. reciprocal of the last dilution of the serum sample in which the number of PCV2 infected cells was reduced to a 50%) was calculated and designated as the neutralizing antibody (NA) titre. Results were expressed as log_2_ NA titre.

### PCV2 amplification and sequencing

With the aim of determining the main PCV2 genotype circulating in the farm, the capsid protein gene (ORF2) was sequenced from two PCV2 qPCR positive samples per treatment group. Amplification was done from nucleotide 1050 to 1735 (PCV2 genome; GenBank Accession Number: AY181948) using primers PCV2all_F (5′ GGGTCTTTAAGATTAAATYC 3′) and PCV2all_R (5′ ATGACGTATCCAAGGAG 3′). PCR was developed in a 25 μL reaction containing 1.25 μL of each mentioned primer at 10 pmol/μL, 5 μL of 5 × PCR buffer, 2.5 μL of MgCl_2_ at 25 mM, 0.75 U of Taq DNA polymerase, 1 μL of dNTP stock solution at 5 mM, 11.35 μL of DEPC-treated water and 2.5 μL of extracted DNA. The PCR was started with an initial denaturation step of 5 min at 94 °C. The temperature profile of the following 40 cycles consisted of 30 s at 95 °C for denaturation, 30 s at 53 °C for primer annealing and 40 s at 72 °C for elongation. The reaction was terminated by a final elongation step of 7 min at 72 °C. Amplified PCR product was run in an electrophoresis gel with 1.8% agarose. The band was purified using NucleoSpin Gel and PCR Clean-up kit (Macherey–Nagel, GmbH & Co. KG, Germany) according to the manufacturer’s instruction. Sequencing reactions were performed with BigDye Terminator v3.1 Cycle Sequencing Kit (Applied Biosystems, Foster City, CA, USA) and analysed using a 3130 × l Genetic Analyser (Applied Biosystems).

### PCV2 capsid protein (ORF2) phylogenetic and sequence analysis

Nucleotide sequences of the PCV2 capsid protein were analysed using Bioedit v7.0.9.0 [25]. Sequences were aligned using the Clustal W multiple alignment method included in the Bioedit package [26]. Fifteen strains of different PCV2 genotypes retrieved from the GenBank database were included in the comparison. The phylogenetic tree was constructed according to the Neighbor-Joining method with 1000 bootstrap replicates using MEGA version 4 [27].

### Statistical analyses

Animal mortality and exclusion rates between groups were compared using the likelihood ratio test. Generalized linear mixed models for longitudinal binary data were performed to analyse the evolution between groups for PCV2 qPCR (positive/negative) values in pigs (serum samples) and pens (OF samples). Treatment group, sampling point and their interaction were considered as fixed effects, and piglet and pen as random effects. Whenever differences between groups were detected, they were further evaluated by pairwise comparisons. *p*-values were corrected using Tukey’s method. Generalized linear mixed models were applied for longitudinal continuous data such as mean log_10_ PCV2 copies/mL in qPCR positive serum and OF samples, mean ELISA S/P IgG values in sera, mean ELISA semi-quantitative values in OF and log_2_ NA titre in sera. The comparison of PCV2 AUC load in serum samples between groups was analysed by a non-parametric test (Kruskall-Wallis statistic). Pearson’s correlation coefficient was used to assess the relationship between serum and OF results (ELISA and qPCR), as well as between ELISA values from both serum and OF samples in comparison to NA titres in serum samples. Statistical analyses were carried out using SAS v9.4, SAS Institute Inc., Cary, NC, USA. The significance level was set at *p* < 0.05.

## Results

### Clinical signs and mortality

No clinical signs related to PCV2-SD were observed during the course of the study. No statistically significant differences in terms of mortality rate and animal exclusion (ranging from 2.5 to 7.5% in all groups) were observed among treatment groups during the whole experimental period (data not shown).

### Quantification of PCV2 DNA

#### Serum samples

**Figure 1:**
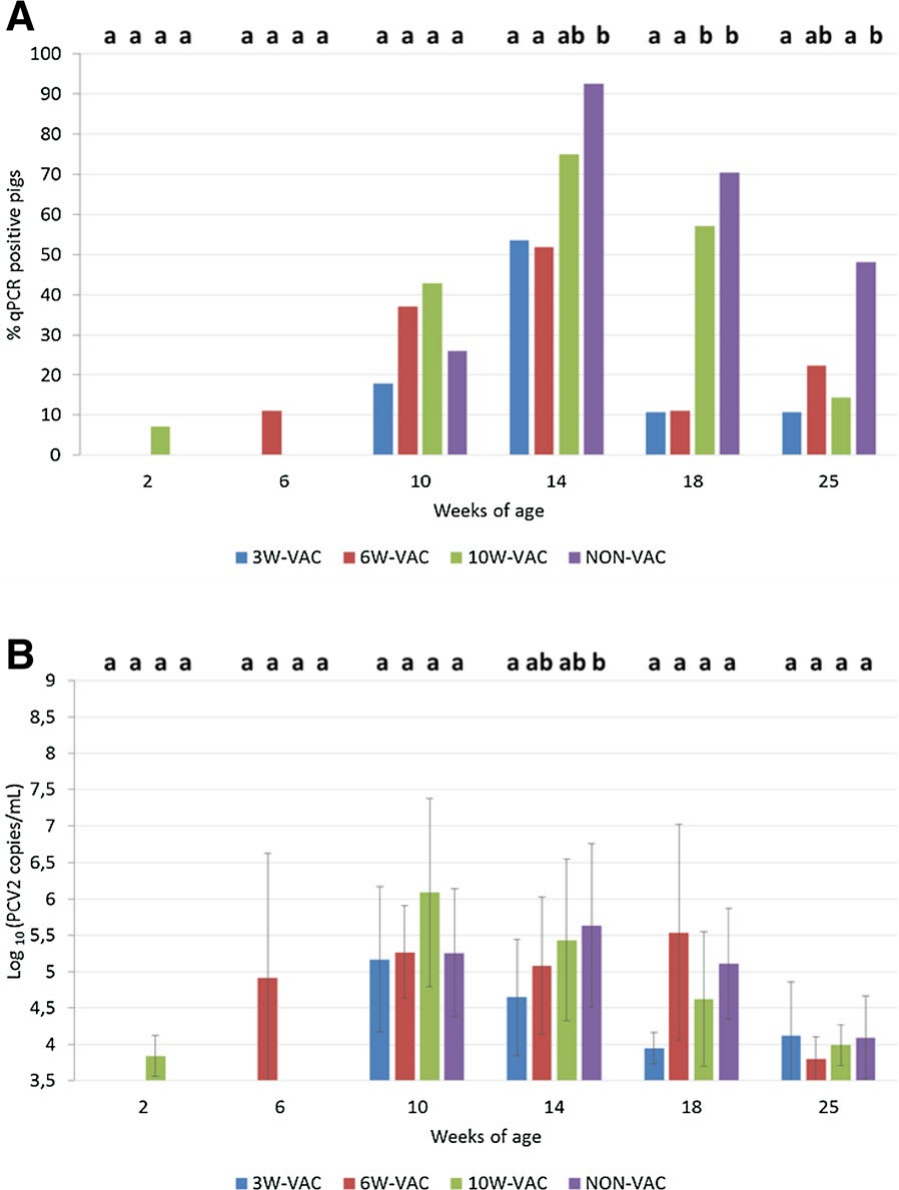
**Percentage of PCV2 qPCR positive pigs (A) and mean viral load (±SD) of qPCR positive serum samples (B).** Different letters in superscript mean statistically significant differences (*p* < 0.05) among experimental groups at each sampling point.

While very few pigs were qPCR positive at 3 and 6 weeks of age, the percentage of PCV2-DNA positive pig sera raised at 10 weeks of age, was maximum at 14 weeks of age and then started to decrease by 18 and 25 weeks of age. Particularly, animals from 3W-VAC to 6W-VAC groups had a significantly lower (*p* < 0.05) percentage of viremic animals (Figure 1A) compared to the NON-VAC group at 14, 18 and 25 weeks of age (in 3W-VAC group) and at 14 and 18 weeks of age (in 6W-VAC group). In contrast, the 10W-VAC group showed a higher percentage of viremic pigs than 3W-VAC and 6W-VAC groups at 10, 14 and 18 weeks of age (only significantly different at 18 weeks of age), but lower than that of control group at 14, 18 and 25 weeks of age (only significant at 25 weeks of age). At the peak of infection (14 weeks of age), the 3W-VAC group showed a significantly lower (*p* < 0.05) PCV2 load (Figure 1B) than the NON-VAC group. The 6W-VAC and 10W-VAC groups also showed the same trend with lower viral loads than the NON-VAC group but these differences were not statistically significant. The 3W-VAC and 6W-VAC groups experienced a significantly lower AUC of viral load than the NON-VAC group (*p* < 0.05) (Table 2). However, PCV2 AUC of 10W-VAC group was only numerically lower than that of the NON-VAC group.

**Table 2:** **Area under the curve (AUC) of PCV2 load (log**_**10**_ **PCV2 copies/mL) in serum samples from 2 to 25** **weeks of age**

Group	Mean AUC of viral load
**3** **W-VAC**	3.25^a^ (Min. = 0.00/Max. = 6.48)
**6** **W-VAC**	3.79^a^ (Min. = 0.00/Max. = 7.41)
**10** **W-VAC**	4.92^ab^ (Min. = 0.00/Max. = 7.75)
**NON-VAC**	5.65^b^ (Min. = 0.00/Max. = 7.90)

Different letters in superscript mean statistically significant differences (*p* < 0.05) among experimental groups.

#### OF samples

**Figure 2:**
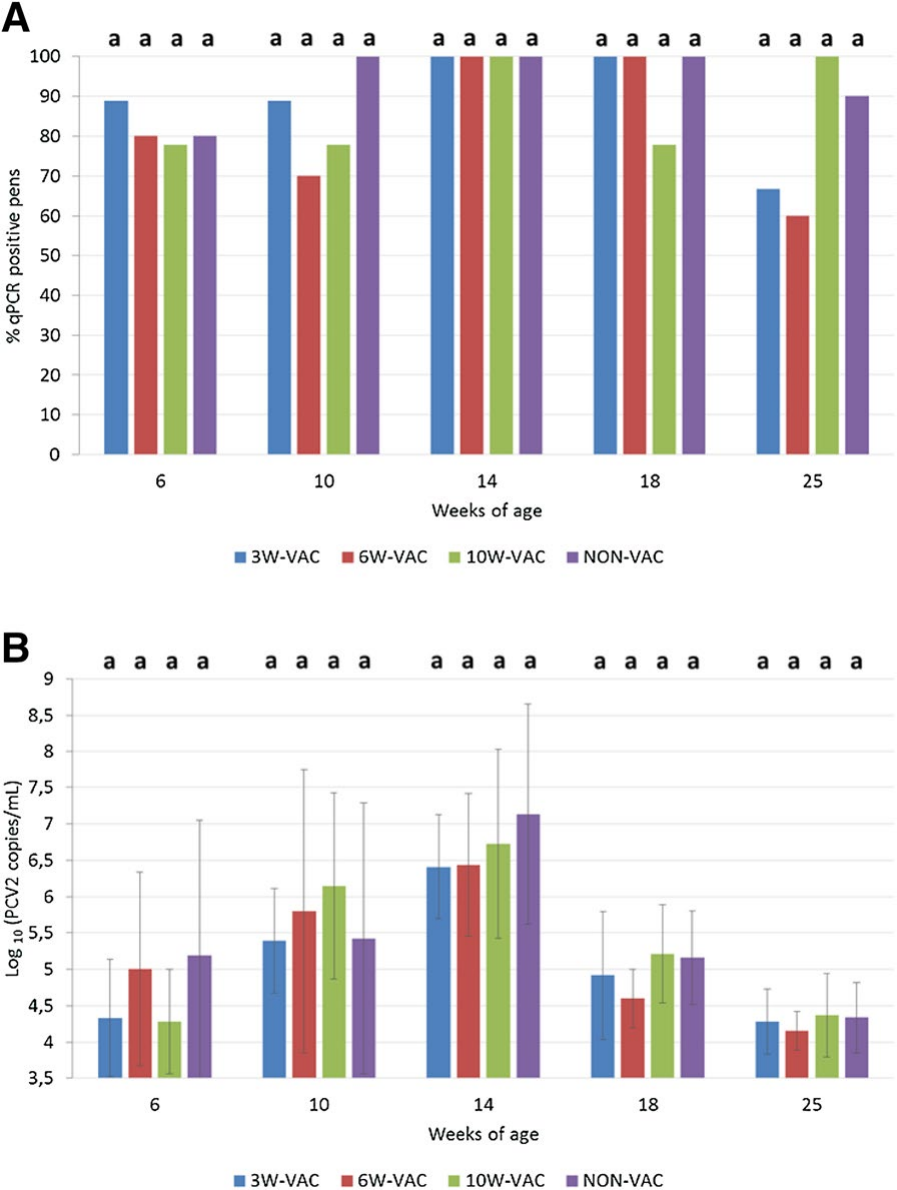
**Percentage of PCV2 qPCR positive pens (A) and mean viral load (±SD) of qPCR positive oral fluids samples (B).**  Different letters in superscript mean statistically significant differences (*p* < 0.05) among experimental groups at each sampling point.

At 3 weeks of age, OF collection was not possible since piglets did not chew the ropes. Percentage of PCV2 qPCR positive pens (Figure 2A) was high at all sampling points and no statistically significant differences among treatment groups were observed. At the peak of infection (14 weeks of age), 100% of positivity was observed in all groups. At this time point, viral load (Figure 2B) was numerically lower in all the vaccinated groups compared to the control group.

Virological results obtained from serum and OF samples showed positive but non-significant correlations: percentage of PCV2 qPCR positive samples (*r* = 0.86; *p* = 0.06) and viral load (*r* = 0.76; *p* = 0.13).

### Serology

#### Anti-PCV2 IgG antibody levels in serum samples

**Figure 3:**
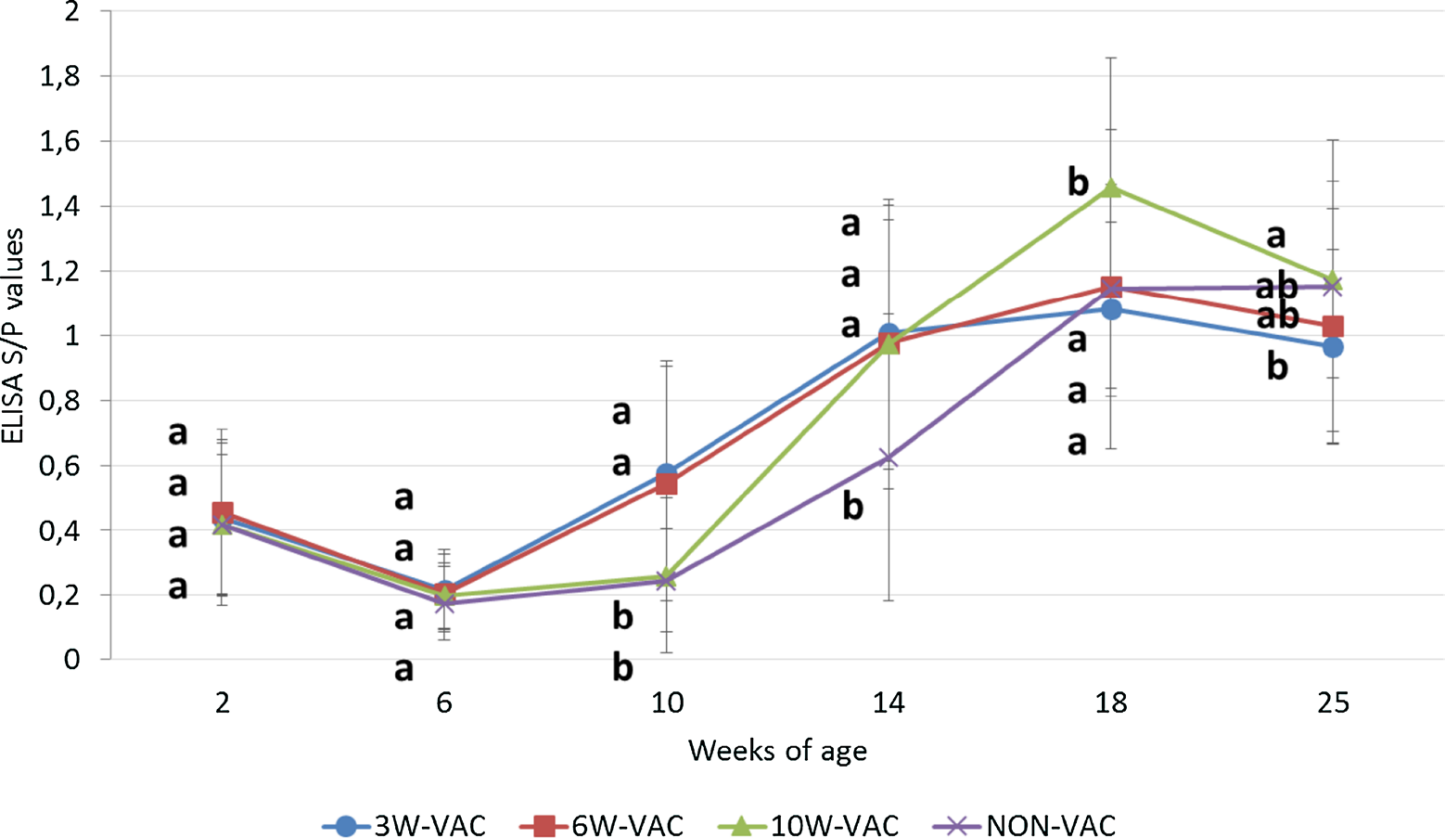
**PCV2 ELISA S/P results (mean** **±** **SD) in serum samples.** Different letters in superscript mean statistically significant differences (*p* < 0.05) among experimental groups at each sampling point.

The course of antibodies against PCV2 in the four treatment groups is shown in Figure 3. From 2 to 6 weeks of age, all groups presented a decrease of ELISA S/P values and no differences between groups were observed. At 10 weeks of age, 3W-VAC and 6W-VAC groups showed significantly higher (*p* < 0.05) S/P values than 10W-VAC and NON-VAC groups. The 10W-VAC group seroconverted by 14 weeks of age, reaching significantly higher antibody levels at 18 weeks of age compared to the other groups. The NON-VAC group seroconverted by 14 to 18 weeks of age. From this time point onwards, S/P values of the vaccinated groups began to decrease whereas the ones of the control group remained stable.

#### Anti-PCV2 antibody levels in OF samples

**Figure 4:**
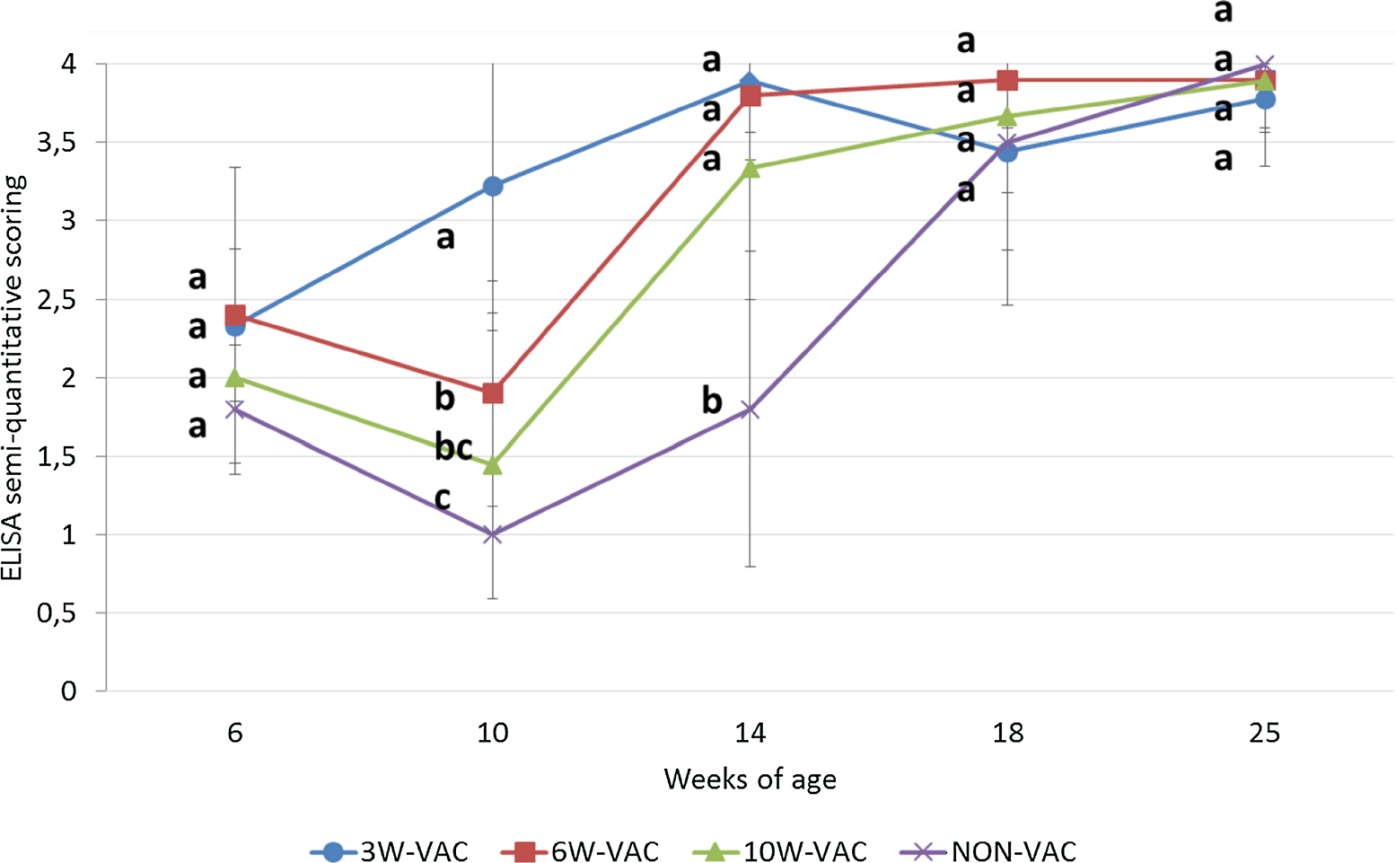
**PCV2 ELISA semi-quantitative values (mean** **±** **SD) in oral fluid samples.** Different letters in superscript mean statistically significant differences (*p* < 0.05) among experimental groups at each sampling point.

Mean semi-quantitative antibody values in OF are summarized in Figure 4. At 10 weeks of age, the 3W-VAC group displayed a statistically significant increase of antibody response compared to all other groups. At the same time point, the other groups experienced a decrease of antibody levels, being the ones of the 6W-VAC group significantly higher than the ones of the NON-VAC group. Four weeks later, i.e. at the peak of infection, all vaccinated groups showed significantly higher antibody values than the NON-VAC group. From 18 weeks of age onwards, antibody levels from all groups remained high and no significant differences were observed between them.

A high and statistically significant correlation (*r* = 0.95, *p* = 0.015) between serum and OF ELISA results was observed.

#### Neutralizing antibody titres in serum samples

**Figure 5:**
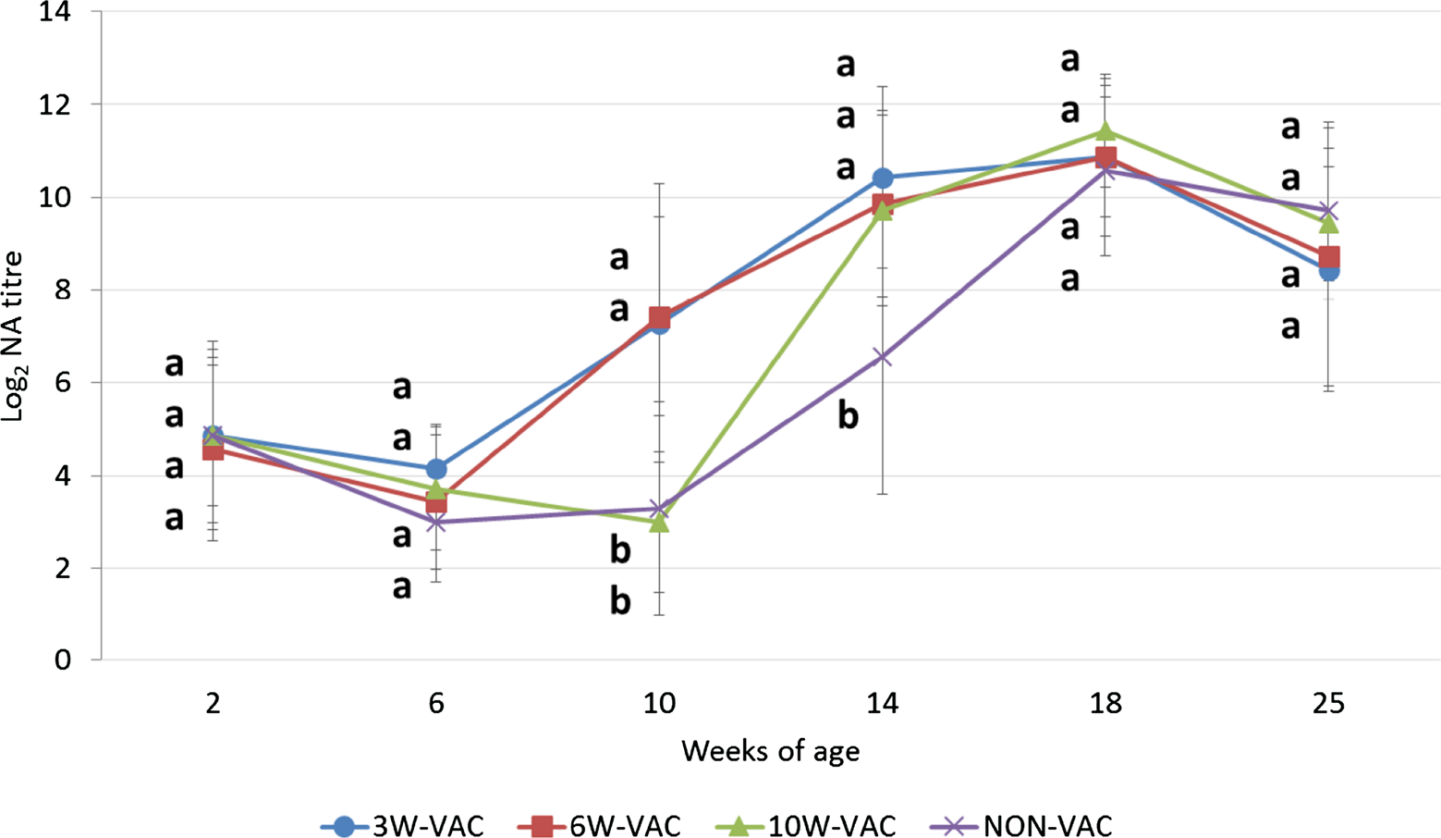
**PCV2 NA titres (mean** **±** **SD) in serum samples.** Different letters in superscript mean statistically significant differences (*p* < 0.05) among experimental groups at each sampling point.

Mean NA titres (± SD) dynamics for each treatment group is depicted in Figure 5. From 2 to 6 weeks of age, all groups showed a decrease of NA titres and no differences between groups were observed. Subsequently, pigs from groups 3W-VAC and 6W-VAC had significantly higher NA levels compared to the 10W-VAC (at 10 weeks of age) and NON-VAC (at 10 and 14 weeks of age) pigs. In the 10W-VAC group, the increase of NA titres was observed 4 weeks after vaccination, i.e. 14 weeks of age, being significantly higher than the ones in NON-VAC pigs. The NA response for animals of the NON-VAC group was detected at 14 weeks of age, reaching maximum levels at 18 weeks of age. After this sampling point, NA levels from all groups began to decrease.

High and statistically significant correlations were found between NA titres tested in serum samples in comparison to ELISA values detected in serum (*r* = 0.97, *p* = 0.001) and OF (*r* = 0.90, *p* = 0.038) samples.

### PCV2 genotyping

**Figure 6:**
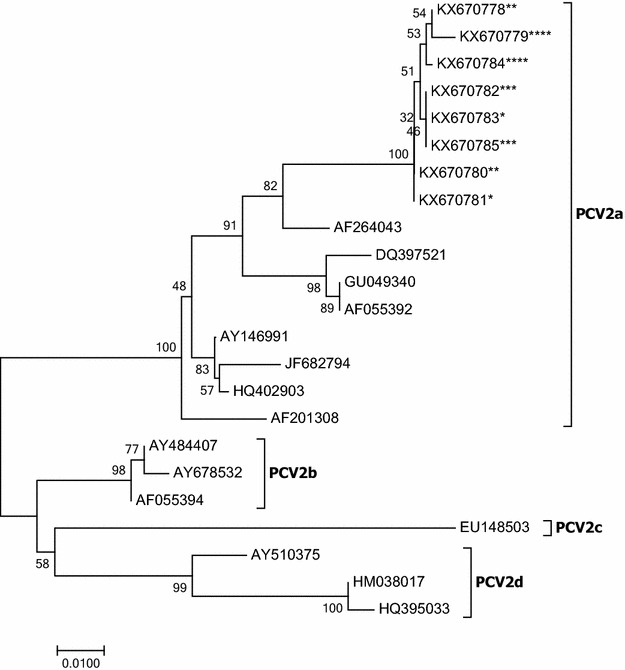
**Neighbor-Joining phylogenetic tree with 1000 bootstrap replicates showing the relationships among the nucleotide sequences of the PCV2 capsid protein.** PCV2 strains sequenced in this study from 3W-VAC (*), 6W-VAC (**), 10W-VAC (***) and NON-VAC (****) groups are compared to PCV2 types a, b, c and d strains. Horizontal branches indicate the sequence distance (number of base differences per site).

A Neighbor-Joining phylogenetic tree including the relationships among the PCV2 isolates sequenced in this study (two per experimental group) and reference strains is represented in Figure 6. All serum samples sequenced (GenBank accession numbers: KX670778–KX670785) were genetically closely related and clustered within PCV2a genotype.

## Discussion

Several studies have shown that PCV2 piglet vaccination at weaning age (3–4 weeks of age) is effective regardless of the PCVD farm status (PCV2-SD or PCV2-SI) and the brand of commercial vaccine used [3]. Vaccination schedules at earlier ages with one single dose are rarely applied, since high levels of maternally derived antibodies (MDA) at the vaccination time may cause a lower humoral immune response (interference with the seroconversion) elicited by the vaccine [6, 11, 28, 29] and may eventually jeopardize the efficacy of vaccination [8, 30].

In the peer-reviewed literature, little information does exist on PCV2 vaccine efficacy obtained by comparing vaccination of piglets (coming from non-vaccinated sows) at different postweaning ages [11, 12]. In the present study, PCV2 vaccination in piglets at 3 or 6 weeks of age yielded similar virological and serological results, producing a relatively early humoral immune response and reducing the proportion of viremic animals in comparison to the unvaccinated group. These results are in accordance with two previously published trials, where no statistically significant differences in terms of PCV2 viremia and/or humoral and cellular immunity were found between pigs vaccinated at 3 and 6 weeks of age [12], and at 3–7 weeks of age [11]. In addition, it has also been demonstrated that vaccination of older animals (8.5 weeks of age) with a subunit vaccine and under a PCV2-SD scenario resulted in a significantly lower mortality in vaccinates than in controls [31]. Although in this latter study, serological and virological parameters from pigs vaccinated at this time point were not tested, the vaccination took place, most probably, when a proportion of pigs were already infected. As far as the authors knowledge, no more information is available on the efficacy of piglet vaccination at older ages. Thus, the present study represents the first time comparing the use of vaccination at 10 weeks of age (entering to the fattening facilities) with earlier ages. Under the conditions of the present farm trial, PCV2 vaccination at 10 weeks of age was probably done too late for an optimal performance as it coincided with the increase of the percentage of viremic pigs in all the treatment groups. Under field conditions, PCV2 viremia usually starts at the end of the nursery or at the beginning of fattening periods, although it is variable depending on the farm and even the production batch [32]. In the present farm scenario, vaccination at 10 weeks of age was able to numerically reduce the percentage of viremic animals at 14 (peak of infection), 18 and 25 weeks of age in comparison with the control group, being statistically significant at the latter time point. This evidence is in agreement with a previous experimental trial [33], showing that vaccination of PCV2 viremic and seropositive piglets leads to a humoral and cellular immune response able to reduce PCV2 viremia. Therefore, although not optimal, vaccination of viremic pigs seems to exert a positive effect compared to viremic, non-vaccinated ones.

The current work further demonstrated the ability of an inactivated vaccine to produce a NA response after piglet immunization at different ages. This response led to a significantly greater protection (in terms of PCV2 viremia) of groups vaccinated before natural infection compared to the group vaccinated after the onset of infection, i.e. at 10 weeks of age, and the control group. The inverse dynamics between NA titres and PCV2 load in serum found in the present study had previously been described [24, 34, 35]. In addition, an immune response analysis of the four major vaccines available on the market was performed in a recent study [36], confirming the ability of the inactivated vaccine used in this study to induce a NA response after vaccination, producing higher NA levels than the ones from subunit vaccines. Moreover, in the current work, high and statistically significant correlations were found between ELISA values from both serum and OF samples in comparison to NA titres in sera. This finding suggests that antibody levels tested by the used ELISA kits might be used as potential predictor of NA titres.

Both serological techniques used in serum and OF samples offered similar results with a high and statistically significant correlation among them. These results would suggest that OF samples can be an alternative to serum for studying PCV2 antibody dynamics. This outcome is in agreement with a reported trial [16] in which positive correlation between OF and pooled sera in terms of antibody detection was found. In contrast, a higher PCV2 qPCR positivity was detected in the present study from OF in comparison with sera at all sampling points, and no significant differences between treatment groups were observed by using OF. Moreover, PCV2 circulation was detected earlier in OF (from 6 weeks of age) compared to sera (from 10 weeks of age) in all groups of pigs. These findings are in accordance with a previous study [16] and support the fact that the starting time of PCV2 circulation and viremia might be different. However, in terms of PCV2 load, whereas similar levels of PCV2 DNA in OF and serum samples with a significant correlation were described [16], higher mean viral loads in OF (over one logarithm) with no significant correlation to PCV2 loads in sera were detected in the current study at infection peak (14 weeks of age). The higher qPCR positivity percentages and PCV2 load in OF compared to sera may be explained by a number of reasons. First, serum samples were obtained from only two or four pigs per pen, but OF sample was a collective sample representing around 23 or 11 pigs per nursery or fattening pen, respectively. Therefore, there is a reasonable probability that some viremic/shedder animals were not bled or alternatively that the bled subjects were not the ones with the highest viral loads. Second, since PCV2 replicates firstly in the tonsil [37, 38], it might be probably detected at an earlier stage and with a greater concentration in OF with regards to sera as has been previously suggested [16]. Finally, PCV2 is an endemic and very stable virus [39] that might be ever-present in pens [40]. In fact, it has been demonstrated that PCV2 subclinically infected pigs may excrete medium to high viral loads in faeces [41, 42]. Therefore, it should be taken into account that ropes might be spoiled by the traces of faeces present in the mouth/skin of the pigs.

In all sequenced samples (*n* = 8), PCV2a genotype was identified. Although this genotype has a worldwide distribution [43], the most current prevalent genotype in the pig population is PCV2b [1, 44]. Indeed, it has been proposed that PCV2b is more prevalent than PCV2a in PCV2-SD cases and in vaccinated farms [45]. The PCV2-SI scenario in the studied farm and the fact that no PCVD compatible clinical signs had ever been observed before the start of the study (and in consequence, vaccination had never been applied before this trial) might be related with the detection of PCV2a genotype in the farm. The apparent sole presence of PCV2a genotype was not enough to produce overt disease in this farm. In fact, the speculation that PCV2a might not be as efficient as PCV2b to trigger clinically evident disease came from the demonstration of a worldwide genotype shift from PCV2a to PCV2b coinciding with major outbreaks of PCV2-SD around the globe [1].

In conclusion, under the conditions of this study, the optimal age for piglet vaccination was at 3 or 6 weeks of age, since it was applied when the percentage of viremic pigs was minimal, triggering an effective humoral immune response before the peak of infection. These strategies were able to reduce, at different sampling points, the proportion of viremic animals in comparison to the unvaccinated group. In contrast, PCV2 vaccination at 10 weeks of age (coinciding with the increase of the percentage of viremic pigs in the population) only achieved such reduction at 25 weeks of age. Therefore, age at PCV2 vaccination should be adapted according to the viral infection dynamics present in the studied farm. Moreover, both serological techniques used in sera and OF were useful to study PCV2 antibody dynamics. In contrast, viral detection in OF might be useful to have an idea of the infection dynamics at population level but should remain only as a raw indicative method.

## Abbreviations

AUC: area under the curve
DEPC: diethylpyrocarbonate
DMEM: Dulbecco’s Modified Eagle Medium
ELISA: enzyme-linked immunosorbent assay
Ig: immunoglobulin
IPC: internal positive control
MDA: maternally derived antibodies
NA: neutralizing antibodies
OD: optical density
OF: oral fluid
PCR: polymerase chain reaction
PCV2: porcine circovirus type 2
PCV2-SD: PCV2 systemic disease
PCV2-SI: PCV2 subclinical infection
PCVD: porcine circovirus disease
PRRSV: porcine reproductive and respiratory syndrome virus
qPCR: quantitative PCR
S/P: sample to positive
SD: standard deviation
VNT: viral neutralization test
%VN: percentage of virus neutralization
